# Spotlighting the need for integrated health information technologies and databases to combat infections and sepsis in long-term care facilities

**DOI:** 10.1093/geroni/igaf134

**Published:** 2025-12-10

**Authors:** Yu Jin Kang, Karen Nielsen, Jingyu Liu

**Affiliations:** Byrdine F. Lewis School of Nursing and Health Professions, Georgia State University, Atlanta, Georgia, United States; School of Public Health, Georgia State University, Atlanta, Georgia, United States; College of Arts and Sciences, Georgia State University, Atlanta, Georgia, United States

**Keywords:** Health Information Technology, Data Curation, Data Integration, Predictive Algorithms, Infection Control

Infections pose a significant threat to long-term care (LTC) facility residents, contributing disproportionately to sepsis, hospitalization, and mortality compared to the general population.^1-3^ As the number of older adults requiring post-acute care continues to rise,^4^ their complex clinical conditions, characterized by multiple morbidities, cognitive impairment, frailty, and pathogen colonization, intensify challenges for infection prevention and control (IPC) practice in LTC facilities.^2^^,^^3^

Systematic IPC programs to aid early identification of sepsis are critical for this vulnerable population, particularly through systematic monitoring of changes in clinical, cognitive, and functional phenotypes across different residents.^2^ However, LTC facilities face significant clinical, technological, and data infrastructure gaps, including a shortage of trained staff with specialized skillsets, inadequate supplies of personal protective equipment, and limited onsite lab capacity for specimen collection and rapid results.^2^ Underdeveloped or inconsistently implemented health information technology (HIT) systems further hinder consistent data collection and analysis needed for infection management, early detection of warning signs, and development of prediction models to support timely sepsis response.^5^

## Opportunities and limitations of long-term care health information technology

Early detection tools can significantly improve sepsis-related outcomes among nursing home residents. For example, a case study implementing an electronic health records (EHR)-based sepsis screening tool showed a substantial increase in detection rates and reduced hospital transfers.^6^ The long-term care data cooperative, which aims to promote comparative-effectiveness research through aggregated LTC EHR, represents another promising step forward.^7^ This initiative creates opportunities to leverage routinely collected resident health data for improving clinical practice and developing decision support tools tailored specifically for LTC environments.^7^

However, current LTC EHR data present several limitations that hinder real-time detection of changes associated with infections and sepsis. These include sparse and low-quality data, limited access to provider narrative documentation, and significant variations in cardiometabolic indicator documentation across facilities and individual residents.^5^^,^^7^^,^^8^ Such irregularity and sparsity impede the ability to identify key changes in a resident’s clinical, mental, and functional status and hinder the development of detection tools such as predictive algorithms based solely on LTC EHR data.^5^^,^^7^

## Leveraging acute care data: an alternative pathway

To address these limitations, an alternative approach involves analyzing richer acute care hospital EHR data from patients admitted from LTC settings.^7^^,^^8^ Hospital data offers a higher frequency and greater clinical detail, providing valuable insights into the progression and characteristics of severe infections in this population. Information from hospital stays can significantly enhance our understanding of sepsis phenotypes and trajectories among LTC residents, enabling comprehensive and systematic care delivery.

For example, when LTC resident transfer records were matched to the hospital EHR, residents presenting with “dyspnea, fever, and hypotension” or “labored breathing and confusion” were frequently diagnosed with sepsis following admission.^8^ These findings illustrate how clinical details from acute hospital records can identify sepsis phenotypes that LTC data alone would miss. Such a comprehensive understanding can inform the development of prediction algorithms for early identification of infections and sepsis tailored to the LTC context. Moreover, this knowledge can highlight which specific clinical data domains are most critical for monitoring resident deterioration and should be prioritized for collection within LTC facilities.

## Toward an integrated data ecosystem

A fundamental barrier to advancing accurate prediction capabilities is the largely segregated data ecosystems between LTC facilities and acute care hospitals. Supplementing LTC records with hospital stay records can both overcome data gaps and provide ground-truth sepsis outcomes for training predictive models.^7^ Despite the compelling need for establishing a broader, interconnected data ecosystem, achieving interoperability across HIT systems appears to be a major challenge to ensuring informational continuity of care. Establishing interoperable HIT systems requires efforts to digitalize paper- and verbal-based information exchange, adoption of standardized clinical ontologies across systems, and infrastructure to ensure patient health information protection.^9^^,^^10^ A recent study developed a broader health information network across HIT systems, showing potential for standardizing and integrating databases but also highlighting challenges with matching information exchange records.^9^ Stronger collaborations and sustained financial support for building network databases and interoperable HIT systems will be essential to move these efforts forward.

## Conclusion: innovation through collaboration

Improving infection management and early sepsis detection in LTC facilities requires both innovation and collaboration across healthcare settings. While the LTC Data Cooperative initiative represents significant progress, its limitations highlight the need for a broader, integrated data strategy. Digitalization of information exchange, protection of patient privacy and information security, adoption of standardized ontology among providers, and development of broader health information networks are necessary to achieve this goal. Continued work in integrating acute care and LTC data is a critical first step toward a more proactive, informed approach to improving IPC and mitigating the impact of infections and sepsis in low-resourced settings.

## Funding

This research was, in part, funded by the National Institutes of Health (NIH) Agreement No. 1OT2OD032581 [AIM-AHEAD] and Byrdine F. Lewis School of Nursing and Health Professions, Georgia State University. The views and conclusions contained in this document are those of the authors and should not be interpreted as representing the official policies, either expressed or implied, of the NIH.

## Conflict of interest

None declared.

## Data Availability

Data sharing is not applicable to this article as no new data were created or analyzed.
